# Chrysin 7‐*O*‐β‐d‐glucopyranoside increases hepatic low‐density lipoprotein receptor expression through AMP‐activated protein kinase activation

**DOI:** 10.1002/2211-5463.13665

**Published:** 2023-07-03

**Authors:** Naoki Kaname, Takahiro Fujimaki, Shotaro Horikoshi, Kaito Fujimura, Manami Kodaka, Shinnosuke Wakamori, Ryo Katsuta, Ken Ishigami, Tsukasa Suzuki, Yuji Yamamoto, Jun Inoue

**Affiliations:** ^1^ Department of Agricultural Chemistry, Faculty of Applied Biosciences Tokyo University of Agriculture Japan; ^2^ Department of Chemistry for Life Sciences and Agriculture, Faculty of Life Sciences Tokyo University of Agriculture Japan

**Keywords:** AMPK, chrysin, LDL receptor, polyphenols, sweet cherry

## Abstract

Elevated plasma low‐density lipoprotein (LDL) cholesterol level is a risk factor for developing atherosclerosis. Increased LDL receptor (LDLR) expression is expected to reduce the risk of atherosclerotic disease since hepatic LDLR is essential for clearing plasma LDL cholesterol. Here, we screened human *LDLR* promoter effectors and observed that extracts from peduncles of sweet cherry (*Prunus avium*) ‘Sato‐Nishiki’ induce *LDLR* gene promoter activity. We used several analytical and chemical methods to show that chrysin 7‐*O*‐β‐d‐glucopyranoside (chrysin‐7G) is one of the compounds that stimulate *LDLR* gene promoter activity in cherry peduncle extracts. Furthermore, synthetic chrysin‐7G increased the expression and activity of LDLR. The chrysin‐7G–mediated increase in LDLR expression and activity was completely abolished by treatment with an AMP‐activated protein kinase (AMPK) inhibitor, compound C. These results indicate that chrysin‐7G increases LDLR expression through AMPK activation and may be a useful compound that can be recycled from waste parts of agricultural products.

## Graphical abstract

Increased LDL receptor (LDLR) expression is expected to reduce the risk of atherosclerotic disease. Here, we screened human *LDLR* promoter effectors and observed that extracts from peduncles of sweet cherry (*Prunus avium*) ‘Sato‐Nishiki’ induce *LDLR* gene promoter activity through AMPK activation. Chrysin‐7G may be a useful compound that can be recycled from waste parts of agricultural products.

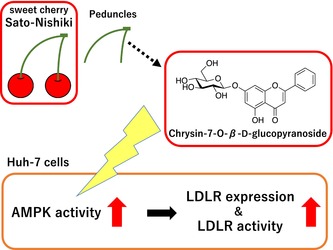

**Abbreviations**
AMPK: AMP‐activated protein kinase
C/EBPβ: CCAAT enhancer‐binding protein‐beta
chrysin‐7G: chrysin 7‐*O*‐β‐d‐glucopyranoside
DMEM: Dulbecco's modified Eagle's medium
DMSO: dimethylsulfoxide
Egr1: early growth response gene 1
EtOAc: ethyl acetate
FAS: fatty acid synthase
GAPDH: glyceraldehyde‐3‐phosphate dehydrogenase
LDH: lactate dehydrogenase
LDL: low‐density lipoprotein
LDLR: LDL receptor
LPDS: lipoprotein‐deficient serum
PCSK9: proprotein convertase subtilisin/kexin type 9
SEM: standard error of the mean
Sp1: specificity protein 1
SREBP‐2: sterol regulatory element‐binding protein‐2

Cardiovascular disorders are the leading causes of death worldwide. Elevated low‐density lipoprotein (LDL) cholesterol level in plasma is a potent risk factor for atherosclerosis and coronary heart disease [1]. The LDL receptor (LDLR) is primarily expressed in the liver, and the liver metabolizes plasma LDL through LDLR‐mediated endocytosis [2]. High LDLR expression in the liver suppresses plasma LDL‐cholesterol levels and is believed to be effective for preventing atherosclerotic diseases.

LDLR expression is regulated at various levels, including transcription, mRNA degradation, post‐translational modifications, and protein degradation [3, 4, 5, 6]. The most widely known *LDLR* gene transcription regulation is by sterol regulatory element‐binding protein‐2 (SREBP‐2), which is activated by decreased intracellular cholesterol levels [7]. The interaction between SREBP‐2 and specificity protein 1 (Sp1) on the *LDLR* gene promoter region is required for normal *LDLR* promoter sterol regulation [8]. Moreover, to activate the *LDLR* gene promoter, estrogen receptor α forms a complex with Sp1 and binds to the promoter region [9]. Additionally, early growth response gene 1 (Egr1) and CCAAT enhancer‐binding protein‐beta (C/EBPβ) are involved in the activation of the *LDLR* gene promoter by oncostatin M [10]. Proprotein convertase subtilisin/kexin type 9 (PCSK9) expression is induced by SREBP‐2 and binds to the LDLR extracellular domain and alters LDLR protein trafficking, thereby promoting its lysosomal degradation [11, 12, 13].

Chrysin is a flavonoid, classified as a flavone, and is found in honey, propolis, and various plants [14]. Chrysin exhibits several pharmacologic activities against cancer, diabetes, cardiovascular diseases, and neurodegenerative diseases [15, 16]. Furthermore, chrysin is found in plants as glycosides; however, whether it is effective in glycoside form is not fully understood [14].

We have previously reported that small compounds of food ingredients increase LDLR expression using Huh‐7 cells that stably express a luciferase reporter and are driven by the *LDLR* gene promoter [17, 18]. In the present study, we used this cell line to identify compounds that stimulate the *LDLR* gene promoter activity from waste parts of agricultural products.

## Results

### Cherry peduncles contain compounds that stimulate the *LDLR* gene promoter activity

**Fig. 1:**
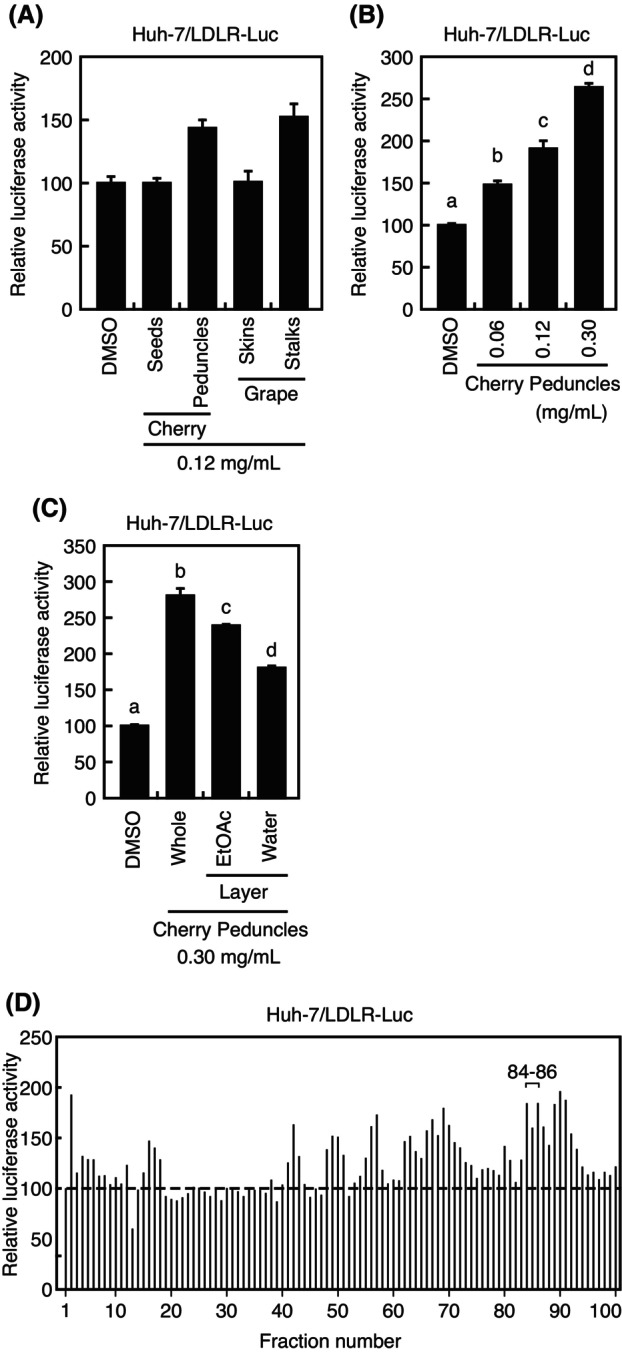
Effect of extracts from waste parts of agricultural products on the low‐density lipoprotein receptor (*LDLR*) gene promoter activity. (A–D) Huh‐7/LDLR‐Luc cells were treated with the indicated extract or the vehicle (DMSO) for 24 h, and luciferase assays were performed. Promoter activity in the presence of DMSO is represented as 100%. (A–C) All data are expressed as mean ± SEM (*n* = 3). Different superscript letters denote statistical significance (*P* < 0.05) (one‐way analysis of variance followed by Tukey's *post‐hoc* test). (D) The experiment is conducted with *n* = 1. Similar results are obtained in two separate experiments.

We have previously established a stable cell line that expresses the luciferase reporter gene under the control of the *LDLR* promoter region from −595 to +36 [17]. Using this stable cell line, we aimed to identify compounds that increase LDLR expression from waste parts of agricultural products, including cherry seeds, cherry peduncles, grape skins, and grape stalks. 50% ethanol extracts of cherry peduncles and grape stalks stimulated the *LDLR* gene promoter activity (Fig. 1A). Since grape stalks contain high levels of resveratrol, which we have previously reported to increase LDLR expression [19], our later experiments focused on cherry peduncles. Cherry peduncle extracts stimulated the *LDLR* gene promoter activity in a dose‐dependent manner (Fig. 1B). Next, the cherry peduncle extracts were divided into ethyl acetate (EtOAc) and water fractions. Both fractions stimulated the promoter activity of the *LDLR* gene (Fig. 1C). Although the activating capacity of both fractions was attenuated compared with the extracts before fractionation (whole), the EtOAc fraction stimulated it more than the water fraction, suggesting that the responsible compounds are contained more abundantly in the EtOAc fraction. Therefore, using HPLC we further fractionated the EtOAc fraction into 100 fractions. Several fractions with activation potential are noted, as shown in Fig. 1D. These results indicate that cherry peduncles contain multiple compounds that stimulate the *LDLR* gene promoter activity.

### Chrysin‐7G is one of the compounds that stimulate the *LDLR* gene promoter activity in cherry peduncle extracts

**Fig. 2:**
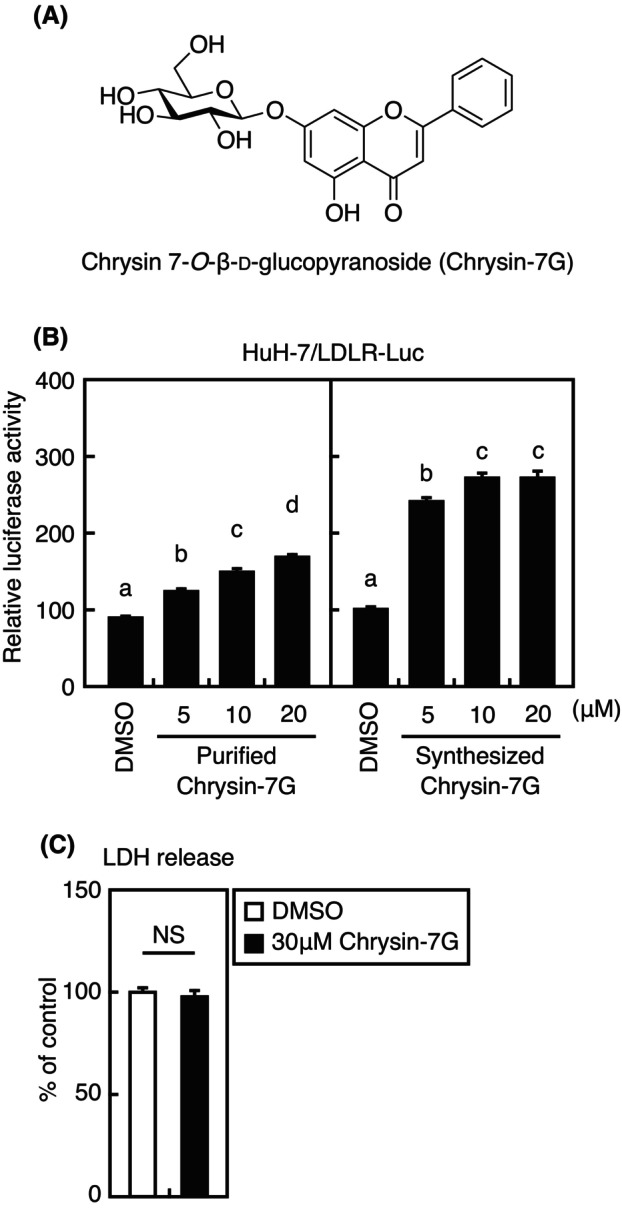
Effect of chrysin‐7G on the LDLR gene promoter activity and LDH release from Huh‐7 cells. (A) Chrysin‐7G structure (B) Huh‐7/LDLR‐Luc cells were treated with purified chrysin‐7G from cherry peduncles (86% purity), synthesized chrysin‐7G, or the vehicle (DMSO) for 24 h, and luciferase assays were performed. Promoter activity in the presence of DMSO is represented as 100%. (C) Huh‐7 cells were treated with 30‐μm chrysin‐7G for 24 h. Afterward, LDH assay was performed. (B, C) All data are expressed as mean ± SEM (*n* = 3). Different superscript letters denote statistical significance (*P* < 0.05) (one‐way analysis of variance followed by Tukey's *post‐hoc* test) (B). NS, not significant (Student's *t*‐test) (C).

**Fig. 3:**
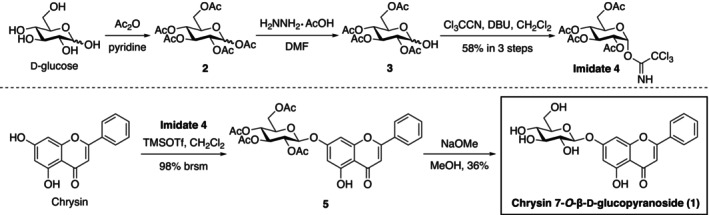
Synthesis of chrysin 7‐*O*‐β‐d‐glucopyranoside (**1**).

Adjacent active fractions were collected and subjected to HPLC analysis to determine the compounds that stimulate the *LDLR* gene promoter activity. Because fractions 84–86 were mainly occupied by a single compound, this peak was used as an indicator to isolate the active compound. NMR analysis of this active compound revealed that chrysin‐7G is the candidate compound (Fig. 2A). Chrysin‐7G, purified at 86% purity from cherry peduncles, stimulated the *LDLR* gene promoter activity (Fig. 2B, left). To further confirm whether chrysin‐7G (1) stimulates the *LDLR* gene promoter activity, 1 was prepared by Schmidt glycosylation employing imidate 4 and commercially available chrysin (Fig. 3). Synthetic chrysin‐7G also stimulated the *LDLR* gene promoter activity, which was stronger than the purified chrysin‐7G (Fig. 2B). These results indicate that chrysin‐7G is the compound that stimulates the *LDLR* gene promoter activity. The synthetic chrysin‐7G was used in later experiments. Next, we investigated the cytotoxic effects of chrysin‐7G on Huh‐7 cells using lactate dehydrogenase (LDH) assay. Treatment with 30‐μm chrysin‐7G for 24 h did not affect the LDH release from Huh‐7 cells (Fig. 2C).

### Chrysin‐7G increases the LDLR expression and activity

**Fig. 4:**
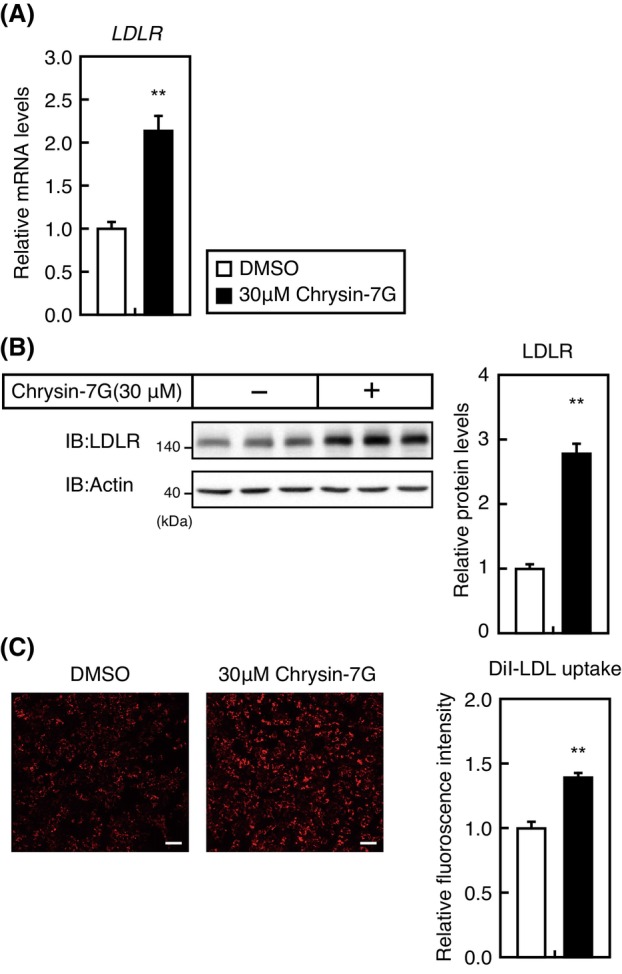
Chrysin‐7G increases the LDLR expression and activity. (A, B) Huh‐7 cells were treated with 30‐μm chrysin‐7G or the vehicle (DMSO) for 24 h; the total RNA and whole‐cell extracts were isolated. (A) Real‐time PCR analysis was performed; mRNA levels were normalized to those of *GAPDH* mRNA and are expressed relative to those in vehicle (DMSO)‐treated controls (*n* = 3). (B) Whole‐cell extracts were subjected to SDS/PAGE and immunoblotting (IB) with anti‐LDLR or anti‐β‐actin antibodies. The signals (*n* = 3) were quantified using image j and normalized by β‐actin signals, and signals of the control group are represented as one. (C) Huh‐7 cells were treated with 30‐μm chrysin‐7G for 24 h followed by culture in a medium supplemented with 10‐μg·mL^−1^ DiI‐labeled LDL for the last 2.5 h. The cells were subsequently examined using fluorescence microscopy. Relative fluorescence levels were quantified using image j, and signals of the control group are represented as one. Scale bar: 100 μm. (A–C) All data are expressed as mean ± SEM (*n* = 3), **P < 0.01 (Student's *t*‐test).

Huh‐7 cells were treated with 30‐μm chrysin‐7G for 24 h to determine the effects of chrysin‐7G on the endogenous LDLR expression. Our real‐time quantitative PCR and immunoblotting analyses demonstrated that LDLR mRNA and protein levels were increased by the treatment with 30‐μm chrysin‐7G (Fig. 4A,B). Therefore, Huh‐7 cells were treated with 30‐μm chrysin‐7G for 24 h and subsequently incubated with the fluorescent‐labeled LDL, DiI‐LDL, for 2.5 h to determine whether treatment with chrysin‐7G causes increased LDL uptake. Subsequent fluorescence microscopy showed increased DiI‐LDL internalization by Huh‐7 cells in the presence of chrysin‐7G (Fig. 4C), suggesting that the chrysin‐7G–mediated increase of LDLR mRNA leads to increased LDL uptake.

### Chrysin‐7G aglycone, chrysin, also stimulates the *LDLR* gene promoter activity and expression at low concentrations

**Fig. 5:**
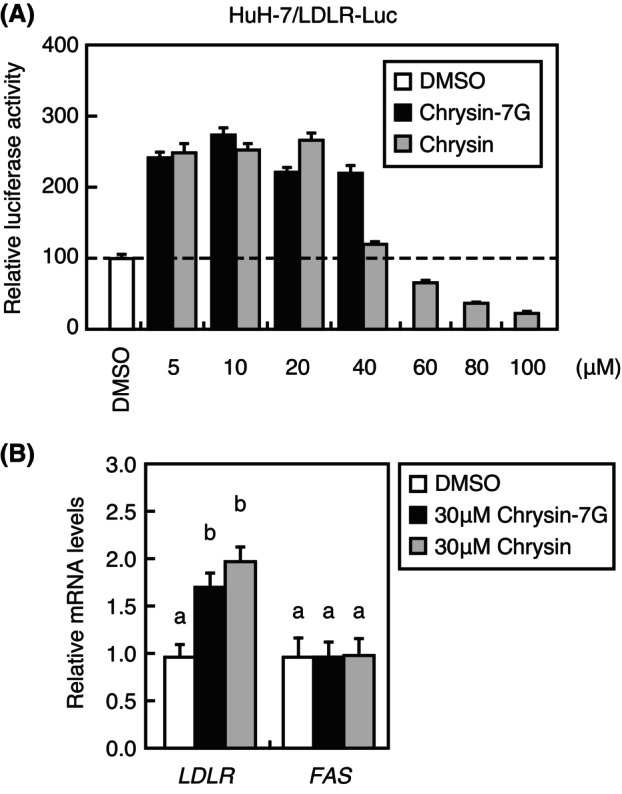
Effects of chrysin‐7G and chrysin on the *LDLR* gene promoter activity and *LDLR* mRNA levels. (A) Huh‐7/LDLR‐Luc cells were treated for 24 h with chrysin‐7G or chrysin at the indicated concentration or the vehicle (DMSO), and luciferase assays were performed. Promoter activity in the presence of DMSO is represented as 100%. (B) Huh‐7 cells were treated for 24 h with chrysin‐7G or chrysin at the indicated concentration or the vehicle (DMSO); total RNA was isolated. Real‐time PCR analysis was performed; mRNA levels were normalized to those of *GAPDH* mRNA and are expressed relative to those in vehicle (DMSO)‐treated controls. (A, B) All data are expressed as mean ± SEM (*n* = 3). Different superscript letters denote statistical significance (*P* < 0.05) (one‐way analysis of variance followed by Tukey's *post‐hoc* test).

Next, we analyzed the effect of chrysin‐7G aglycone, chrysin, on the *LDLR* gene promoter activity and expression. Treatment with 60–100‐μm chrysin decreased the *LDLR* gene promoter activity, whereas treatment with 5–20‐μm chrysin conversely stimulated the activity (Fig. 5A). Treatment with 5–40‐μm chrysin‐7G stimulated the *LDLR* gene promoter activity; however, chrysin‐7G could only be treated up to 40 μm owing to its low solubility (Fig. 5A). Additionally, treatment with 30‐μm chrysin increased *LDLR* mRNA levels in Huh‐7 cells, as well as 30‐μm chrysin‐7G (Fig. 5B). Notably, treatment with 30‐μm chrysin‐7G or chrysin did not increase fatty acid synthase (*FAS*) mRNA levels in Huh‐7 cells (Fig. 5B). These results indicate that chrysin‐7G increases *LDLR* expression even in its aglycones.

### Chrysin‐7G stimulates ERK1/2 phosphorylation; however, ERK pathway activation is not involved in the *LDLR* gene expression stimulation by chrysin‐7G

**Fig. 6:**
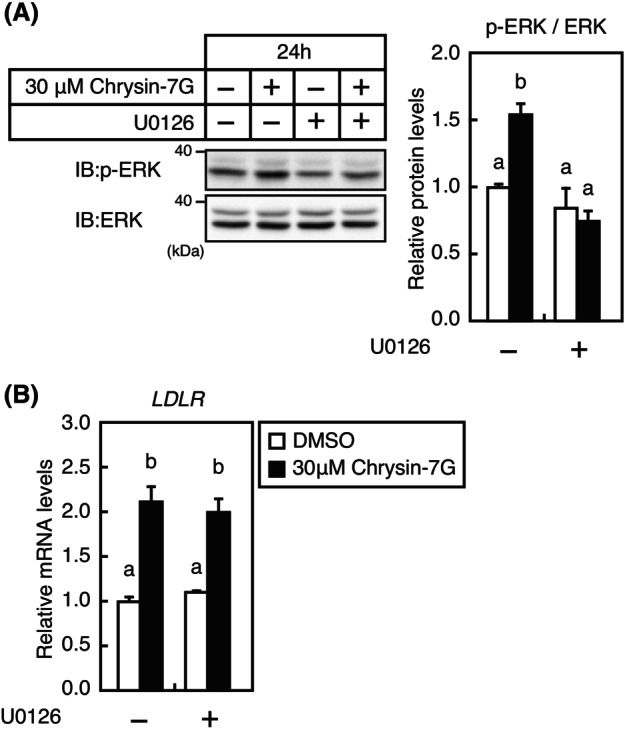
Effect of U0126 on the LDLR mRNA level expression. Huh‐7 cells were treated with 30‐μm chrysin‐7G for 24 h in the presence or absence of 10‐μm U0126; whole‐cell extracts and total RNA were isolated. (A) Whole‐cell extracts were subjected to SDS/PAGE and immunoblotting (IB) with anti‐p‐ERK or anti‐ERK antibodies. Signals (*n* = 3) were quantified using image j and normalized by ERK signals, and the signals of the control group are represented as one. (B) Real‐time PCR analysis was performed; mRNA levels were normalized to those of *GAPDH* mRNA and are expressed relative to those in vehicle (DMSO)‐treated controls. All data are expressed as mean ± SEM (*n* = 3). Different superscript letters denote statistical significance (*P* < 0.05) (one‐way analysis of variance followed by Tukey's *post‐hoc* test).

Next, we examined whether chrysin‐7G treatment stimulates ERK1/2 phosphorylation. As shown in Fig. 6A, 24‐h treatment with chrysin‐7G increases ERK1/2 phosphorylation in Huh‐7 cells. However, chrysin‐7G increased *LDLR* mRNA levels even after the addition of U0126, a specific MEK inhibitor of the ERK pathway (Fig. 6B). These results indicate that the ERK pathway is not involved in the *LDLR* gene expression stimulation by chrysin‐7G.

### AMP‐activated protein kinase is required for chrysin‐7G–mediated stimulation of LDLR expression

**Fig. 7:**
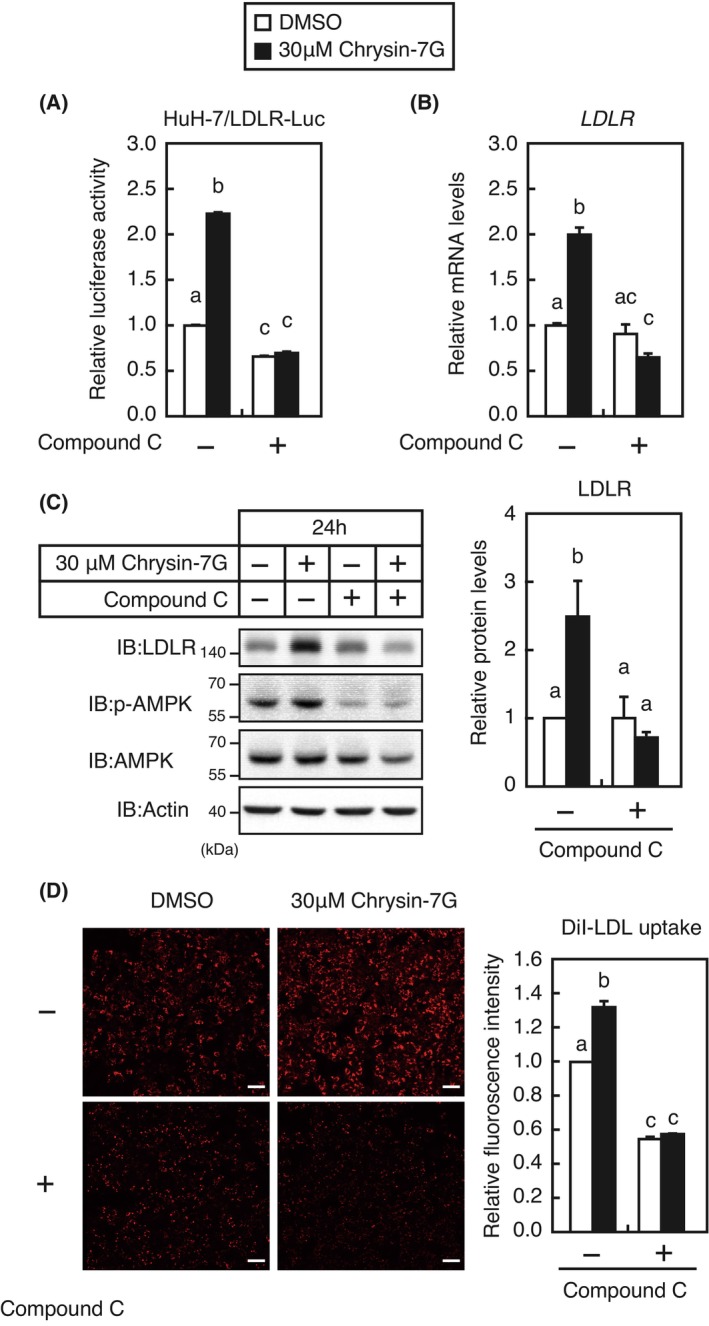
Involvement of AMPK in chrysin‐7G stimulates LDLR expression. (A) Huh‐7/LDLR‐Luc cells were treated with 30‐μm chrysin‐7G for 24 h in the presence or absence of 10‐μm compound C, and luciferase assays were performed. Promoter activity in the presence of DMSO is represented as 100% (*n* = 3). (B–D) Huh‐7 cells were treated with 30‐μm chrysin‐7G for 24 h in the presence or absence of 10‐μm compound C. (B) The total RNA was isolated and real‐time PCR analysis was performed; mRNA levels were normalized to those of *GAPDH* mRNA and are expressed relative to those in vehicle (DMSO)‐treated controls (*n* = 3). (C) Whole‐cell extracts were isolated and subjected to SDS/PAGE and immunoblotting (IB) with anti‐LDLR or anti‐β‐actin antibodies (C), anti‐p‐AMPK, anti‐AMPK, or anti‐β‐actin antibodies. Signals (*n* = 3) were quantified using image j and normalized by β‐actin signals (C), and signals of the control group are represented as one. (D) The cells were cultured in a medium supplemented with 10‐μg·mL^−1^ DiI‐labeled LDL for the last 2.5 h. The cells were subsequently examined using fluorescence microscopy. Relative fluorescence levels were quantified using image j, and signals of the control group are represented as one (*n* = 3). Scale bar: 100 μm. All data are expressed as mean ± SEM (*n* = 3). Different superscript letters denote statistical significance (*P* < 0.05) (one‐way analysis of variance followed by Tukey's *post‐hoc* test).

**Fig. 8:**
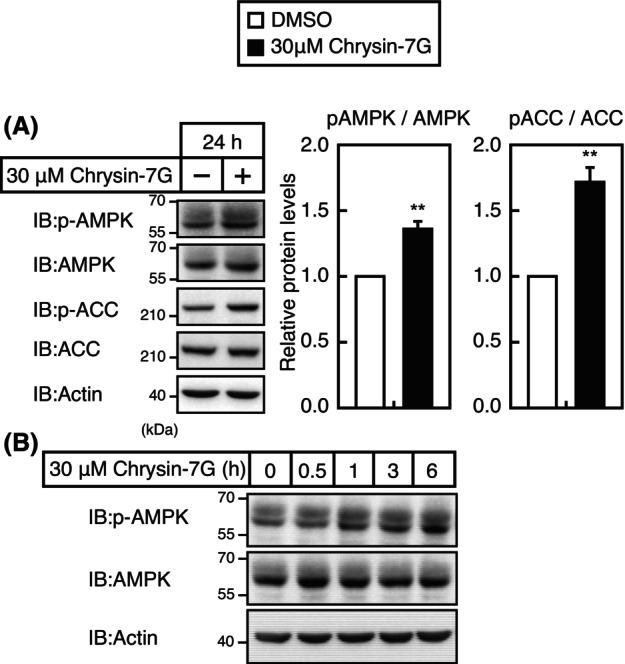
Chrysin‐7G activates the AMPK pathway in Huh‐7 cells. Huh‐7 cells were treated with 30‐μm chrysin‐7G for 24 h (A) or the indicated period (B). Whole‐cell extracts were subjected to SDS/PAGE and immunoblotting (IB) with anti‐p‐AMPK, anti‐AMPK, anti‐p‐ACC, anti‐ACC, or anti‐β‐actin antibodies (A), or anti‐p‐AMPK, anti‐AMPK, or anti‐β‐actin antibodies (B). Signals (*n* = 3) were quantified using image j and normalized by β‐actin signals, and signals of the control group are represented as one. All data are expressed as mean ± SEM (*n* = 3). **P < 0.01 (Student's *t*‐test) (A). Similar results were obtained in two independent experiments (B).

**Fig. 9:**
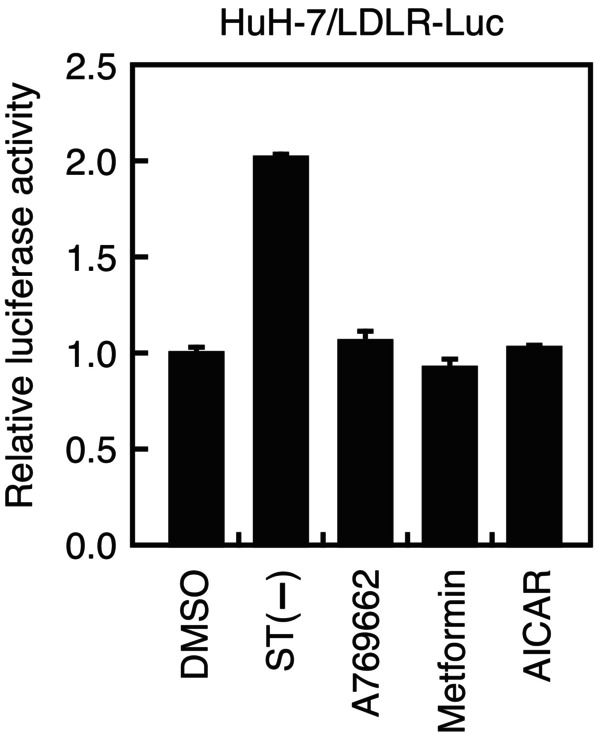
Effect of AMPK activators on the LDLR gene promoter activity. Huh‐7/LDLR‐Luc cells were treated with 50‐μm A769662, 2‐mm metformin, or 1‐mm AICAR or cultured under sterol‐depleted conditions for 24 h [ST(−)], and luciferase assays were performed. Promoter activity in the presence of DMSO is represented as 100% (*n* = 3). All data are expressed as mean ± SEM (*n* = 3).

To explore the signaling pathway by which chrysin‐7G stimulates *LDLR* gene expression, experiments were conducted using inhibitors of several signaling pathways. We observed that treatment with compound C, an AMP‐activated protein kinase (AMPK) inhibitor, completely abolished the upregulation of the *LDLR* gene promoter activity (Fig. 7A) and *LDLR* mRNA and protein levels (Fig. 7B,C) by chrysin‐7G. Treatment with compound C reduced the activity of AMPK, as judged by the reduced phosphorylation levels of AMPK (Fig. 7C). In addition, treatment with compound C completely abolished the upregulation of chrysin‐7G‐mediated LDL uptake (Fig. 7D). The 24‐h treatment with chrysin‐7G significantly increased the phosphorylation levels of AMPK and its substrate ACC (Fig. 8A). Moreover, chrysin‐7G treatment increased the AMPK phosphorylation levels in 1‐ to 6‐h treatment (Fig. 8B). Next, the effect of AMPK activation on the *LDLR* gene promoter activity was examined. The *LDLR* promoter activity was increased under cholesterol‐depleted conditions that stimulate the SREBP activity, although it was unchanged when treated with AMPK activators, A769662, metformin, and AICAR (Fig. 9).

### Chrysin‐7G does not increase the expression levels of *Egr1* and *C/EBPβ* in Huh‐7 cells

**Fig. 10:**
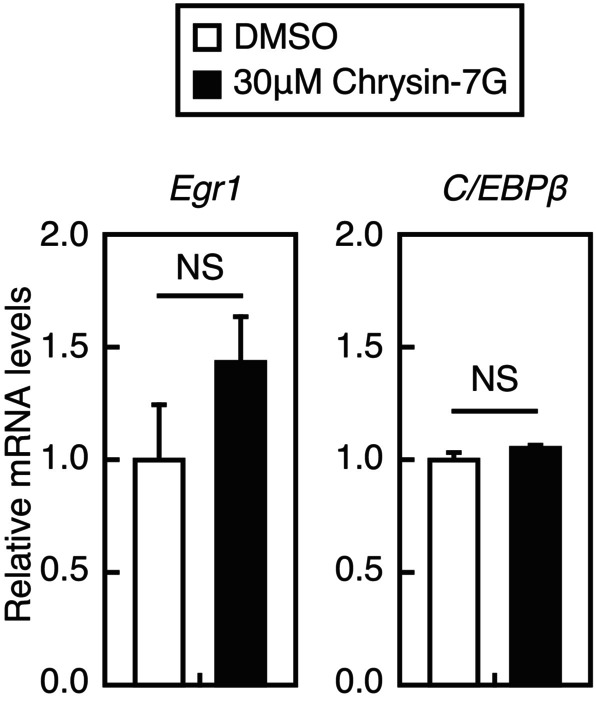
Effect of chrysin‐7G on the expression of Egr1 and C/EBPβ in Huh‐7 cells. Huh‐7 cells were treated with 30‐μm chrysin‐7G for 24 h. The total RNA was isolated and real‐time PCR analysis was performed; mRNA levels were normalized to those of *GAPDH* mRNA and are expressed relative to those in vehicle (DMSO)‐treated controls (*n* = 3). All data are expressed as mean ± SEM (*n* = 3). NS, not significant (Student's *t*‐test).

Because *Egr1* and *C/EBPβ* are involved in the upregulation of *LDLR* expression by oncostatin M [10] and that AMPK activation increases their expression levels [20, 21], we examined the effect of chrysin‐7G on the expression levels of *Egr1* and *C/EBPβ* in Huh‐7 cells. Treatment with chrysin‐7G for 24 h did not stimulate the expression levels of *Egr1* and *C/EBPβ* in Huh‐7 cells (Fig. 10).

## Discussion

In this study, we showed that chrysin‐7G, derived from cherry peduncles, activates LDLR by stimulating the *LDLR* gene promoter activity and that this activation is mediated by the AMPK pathway upregulation.

Agricultural products are significant not only as a source of energy but also as a source of various compounds that affect our health. Our analysis showed that each gram of dry‐weight of cherry peduncles contains 0.35‐mg chrysin‐7G (data not shown). Identification of chrysin‐7G, which increases the LDLR activity, from the disposal parts of cherries, shows a way to effectively use the waste part of cherries and increases its value. In addition to chrysin‐7G, there may be multiple active compounds in the cherry extract that increase the *LDLR* gene promoter activity (Fig. 1C,D). In future, to further increase the added value of cherries, we plan to identify these compounds from cherry peduncles.

In the present study, we showed that the chrysin‐7G aglycone, chrysin, stimulated the *LDLR* gene promoter activity at low concentrations (5–20 μm), whereas high chrysin concentrations (60–100 μm) decreased it (Fig. 5A). We have previously reported that 50–100‐μm chrysin reduced the *FAS* gene expression by suppressing the SREBP activity [22]. Given that the *LDLR* gene promoter is also under the control of SREBP, the reduction of the *LDLR* gene promoter activity at high chrysin concentrations is possibly because of SREBP activity suppression. On the other hand, low chrysin concentrations conversely stimulated the *LDLR* gene promoter activity and expression; however, no change in *FAS* gene expression was noted, suggesting that chrysin acts differently depending on its concentration and that low chrysin concentrations did not suppress the SREBP activity. Considering that low chrysin‐7G and chrysin concentrations have stimulatory effects on LDLR expression, it is likely that these compounds exert their effects from outside the cell at low concentrations since flavonoid glycosides are not readily absorbed into the cell.

It is widely known that plant‐derived compounds, including flavonoids, regulate signaling pathways in mammalian cells. We have previously reported that the flavonoid kaempferol activates the ERK pathway, and this activation leads to the increase in LDLR expression by activating Sp1, whereas other flavonoids, including luteolin and apigenin, also activate the ERK pathway although do not increase LDLR expression [18]. In this study, we demonstrated that chrysin‐7G activates the ERK pathway; however, its activation is not involved in the increase in *LDLR* gene expression. This evidence suggests that ERK pathway activation alone does not upregulate *LDLR* gene expression.

Chrysin, but not chrysin‐7G, has been reported to activate the AMPK pathway in several cell lines [23, 24, 25]. Chrysin‐7G–containing moringa extracts have been reported to inhibit 3T3‐L1 cell differentiation through AMPK activation; however, whether chrysin‐7G is involved in AMPK activation is unclear [26]. In the present study, we showed that synthetic chrysin‐7G activates the AMPK pathway in Huh‐7 cells. Thus, chrysin‐7G may be involved in the AMPK pathway activation by moringa extracts. Moreover, we showed that the AMPK pathway activation by chrysin‐7G leads to increased promoter activity and protein levels of the *LDLR* gene. AMPK activation has been reported to increase LDLR protein levels. AMPK activation downregulates *PCSK9* mRNA levels by suppressing the SREBP activity, thereby stabilizing the LDLR protein [27, 28]. To our knowledge, no reports on AMPK activation increasing the *LDLR* gene promoter activity are available. Considering that several AMPK activators did not stimulate the *LDLR* gene promoter activity (Fig. 7), it appears that chrysin‐7G cannot increase the *LDLR* gene promoter activity by AMPK activation alone. Alternatively, since the strength and persistence of AMPK activation are expected to vary with chrysin‐7G and each AMPK activator, this difference may be responsible for the differential effects of these AMPK activators on LDLR gene promoter activity. Among the transcription factors that activate the *LDLR* gene promoter, it has been reported that AMPK activation suppresses the SREBP‐2 and Sp1 activity [29, 30]; however, it stimulates the Egr1 and C/EBPβ activity by increasing their expression levels in hepatoma cells [20, 21]. Given that chrysin‐7G treatment for 24 h increased *LDLR* expression and activated the AMPK pathway (Figs 4A and 8A) but did not increase the levels of *Egr1* and *C/EBPβ* expression (Fig. 10), *Egr1* and *C/EBPβ* are unlikely involved in the upregulation of chrysin‐7G–mediated *LDLR* expression. Further studies are required to identify transcription factors to increase *LDLR* mRNA levels by chrysin‐7G.

## Materials and methods

### Reagents

Mevalonate, lipoprotein‐deficient serum (LPDS), and U0126 were purchased from Sigma (St. Louis, MO, USA). Blasticidin S, fluvastatin, and AICAR were purchased from Wako (Osaka, Japan). Chrysin, metformin, and A769662 were purchased from Tokyo Chemical Industry (Tokyo, Japan). Compound C (dorsomorphin) was purchased from LC Laboratories (Woburn, MA, USA). DiI‐labeled LDL was purchased from Molecular Probes (Eugene, OR, USA). Chrysin, chrysin 7‐*O*‐β‐d‐glucopyranoside (chrysin‐7G, common name: Chrysin 7‐glucoside, CAS No. 31025‐53‐3), AICAR, A769662, U0126, and compound C were dissolved in dimethylsulfoxide (DMSO), and metformin was dissolved in water. Sweet cherries and grapes were purchased from a local supermarket.

### Media

Medium A contained Dulbecco's modified Eagle's medium (DMEM) supplemented with 100‐U·mL^−1^ penicillin, 100‐μg·mL^−1^ streptomycin, and 10% fetal bovine serum. Medium B contained DMEM supplemented with 100‐U·mL^−1^ penicillin, 100‐μg·mL^−1^ streptomycin, 5% LPDS, 50‐μm sodium mevalonate, and 12.5‐μm fluvastatin.

### Cell culture

Huh‐7/LDLR‐Luc cells were previously established [17] and maintained in medium A containing 2‐μg·mL^−1^ Blasticidin S. Huh‐7 cells were maintained in medium A. All cell cultures were incubated at 37 °C with 5% CO_2_ atmosphere.

### Sample preparation

Dried cherry seeds, cherry peduncles, grape skins, and grape stalks were pulverized in a food mill. Subsequently, each powder was extracted with 10 times the amount of 50% ethanol under reflux conditions. After the filtration, the solvent was removed under reduced pressure to yield a 50% ethanol extract. The extract was dissolved in H_2_O and extracted with EtOAc, and the organic layer was concentrated to yield an EtOAc residue.

### Fraction assay

The EtOAc residue (30 mg) was subjected to HPLC (column: YMC‐Pack ODS‐AQ; 20 mm I.D. × 250 mm; YMC Co., Ltd., Kyoto, Japan) with linear gradient elution from 30% B to 80% B in 65 min (solvent A: 0.1% TFA in H_2_O; solvent B: CH_3_OH) to yield 100 fractions. Each fraction was dissolved in DMSO after drying and subsequently used for the assay.

### Isolation of chrysin‐7G from cherry peduncles

The EtOAc residue (60 mg) was purified by HPLC (column: YMC‐Pack ODS‐AQ; 20 mm I.D. × 250 mm; YMC Co., Ltd.) with a similar method as described above to yield a pale yellow powder (6.3 mg). This powder was further purified by HPLC (column: YMC‐Pack Polymer C18; 10 mm I.D. × 250 mm; YMC Co., Ltd.) with 55% B in 40 min (solvent A: 0.1% trifluoroacetic acid in H_2_O; solvent B: CH_3_OH) to yield chrysin‐7G (0.5 mg).

### Luciferase assays in Huh‐7/LDLR‐Luc cells

Huh‐7/LDLR‐Luc cells were plated on 24‐well plates at a density of 1 × 10^5^ cells/well before culturing in medium A for 24 h; thereafter, these cells were incubated for 24 h, with and without indicated concentrations of extracts of agricultural products, chrysin, or chrysin‐7G. The luciferase activity was measured as previously described [31].

### Synthesis of chrysin 7‐*O*‐β‐d‐glucopyranoside (5‐hydroxy‐2‐phenyl‐7‐(((2*S*,3*R*,4*S*,5*S*,6*R*)‐3,4,5‐trihydroxy‐6‐(hydroxymethyl)tetrahydro‐2*H*‐pyran‐2‐yl)oxy)‐4*H*‐chromen‐4‐one, **1**)

Optical rotations were recorded with a JASCO P‐2100 polarimeter (JASCO, Tokyo, Japan). Melting points were measured with a Yanaco micro‐melting point apparatus (Yanaco, Kyoto, Japan) and are uncorrected values. IR spectra were measured with a JASCO FT/IR‐4100 spectrophotometer. ^1^H and ^13^C NMR were recorded on JEOL‐JNM ECX 400 spectrometer (JEOL, Tokyo, Japan). Chemical shifts (δ p.p.m.) were referenced to the residual solvent peaks as the internal standard (CDCl_3_: δ_H_ = 7.26, δ_C_ = 77.0, DMSO‐*d*_6_: δ_H_ = 2.49, δ_C_ = 39.5). Mass spectra were recorded on a JEOL JMS‐T100LP. Column chromatography was performed using Kanto silica gel 60 N (0.060–0.200 mm).

d‐Glucose (30.0 g, 167 mmol) in dry pyridine (134 mL) was cooled to 0 °C with stirring under Ar atmosphere. After the addition of acetic anhydride (157 mL, 1.67 mol) at 0 °C, the reaction mixture was stirred overnight at room temperature. The reaction was quenched by adding saturated sodium bicarbonate solution, and the mixture was extracted with EtOAc. The combined organic layer was washed with saturated copper(II) sulfate solution and brine successively, dried over anhydrous sodium sulfate, and concentrated *in vacuo* to give crude penta‐*O*‐acetyl‐d‐glucopyranose (**2**, 62.0 g) as a colorless solid, which was used in the next reaction without further purification.

To a solution of hydrazine acetate (1.42 g, 15.4 mmol) in DMF (50 mL) was added pentaacetate **2** (5.0 g, 12.8 mmol) with stirring under Ar atmosphere, and the solution was heated to 50 °C for 1 h. After cooling down to room temperature, the reaction mixture was diluted with water and extracted with EtOAc. The combined organic layer was washed with water and brine successively, dried over anhydrous sodium sulfate, and concentrated *in vacuo* to give crude tetra‐*O*‐acetyl‐d‐glucopyranose (**3**, 3.01 g) as a colorless viscous oil, which was used in the next reaction without further purification.

To a solution of tetraacetate **3** (960 mg) in CH_2_Cl_2_ (55 mL) were added trichloroacetonitrile (1.38 mL, 6.66 mmol) and 1,8‐diazabicyclo[5.4.0]undec‐7‐ene (83 μL, 546 μmol) at 0 °C with stirring under Ar atmosphere. After stirring for 2 h at 0 °C and 2 h at room temperature, the reaction mixture was filtered through silica gel pad. Eluent was concentrated *in vacuo* and the residue was subjected to silica gel column chromatography (Hex/EtOAc = 1 : 1) to give imidate **4** (1.22 g, 58% in 3 steps) as a colorless viscous oil.

Compound **4**: ^1^H NMR (400 MHz, CDCl_3_): δ (p.p.m.) = 8.69 (s, 1H), 6.56 (d, *J* = 3.6 Hz, 1H), 5.57 (t, *J* = 10.0 Hz, 1H), 5.19 (t, *J* = 10.0 Hz, 1H), 5.13 (dd, *J* = 10.0, 3.6 Hz, 1H), 4.28 (dd, *J* = 12.0, 4.0 Hz, 1H), 4.21 (ddd, *J* = 10.0, 4.0, 2.0 Hz, 1H), 4.13 (dd, *J* = 12.0, 2.0 Hz, 1H), 2.08 (s, 3H), 2.05 (s, 3H), 2.04 (s, 3H), 2.02 (s, 3H).

To a solution of the imidate **4** (1.22 g, ca. 2.47 mmol) and chrysin (147 mg, 496 μmol) in CH_2_Cl_2_ (55 mL) was added trimethylsilyl trifluoromethanesulfonate (18 μL, 66 μmol) at 0 °C with stirring under Ar atmosphere. The reaction mixture was stirred for 2 days and kept stirring for further 1 day with additional trimethylsilyl trifluoromethanesulfonate (18 μL, 66 μmol). The reaction was quenched by adding saturated sodium bicarbonate solution, and the separated organic layer was washed with water and brine successively, dried over anhydrous sodium sulfate, and concentrated *in vacuo*. The residue was subjected to silica gel column chromatography (Hex/EtOAc = 5 : 1–1 : 1) to give crude glucoside **5** (141 mg, 49%) as a colorless needle and recover unreacted chrysin (73 mg, 50%).

Compound **5**: ^1^H NMR (400 MHz, CDCl_3_): δ (p.p.m.) = 7.88 (dd, *J* = 8.0, 1.6 Hz, 2H), 7.60–7.50 (m, 3H), 6.70 (s, 1H), 6.61 (d, *J* = 2.4 Hz, 1H), 6.46 (d, *J* = 2.4 Hz, 1H), 5.35–5.28 (m, 2H), 5.21–5.13 (m, 2H), 4.29 (dd, *J* = 12.0, 6.0 Hz, 1H), 4.22 (dd, *J* = 12.0, 2.4 Hz, 1H), 3.95 (ddd, *J* = 10.0, 6.0, 2.4 Hz, 1H), 2.11 (s, 3H), 2.08 (s, 3H), 2.07 (s, 3H), 2.05 (s, 3H).

To a solution of compound **5** (2.44 g, 4.17 mmol) in dry methanol (100 mL) was added sodium methoxide (2.55 g, 41.7 mmol) under Ar atmosphere. After stirring for 2 h at room temperature, the reaction was quenched by adding Amberlite IR120B(H)‐HG, and the mixture was filtered and concentrated *in vacuo*. The residue was recrystallized from methanol/water (2 : 1) to give chrysin‐7G (**1**, 623 mg, 36%) as a pale yellow crystal.

Compound **1**: mp = 202–204 °C. ${\left[\alpha \right]}_{D}^{26}$ = −45 (*c* 1.0, DMF). IR (ATR): ν_max_ (cm^−1^) = 3416, 3232, 2879, 1653, 1611, 1581, 1492, 1376, 1271, 1175, 1070, 1024, 768. ^1^H NMR (400 MHz, DMSO‐*d*_6_): δ (p.p.m.) = 12.8 (s, 1H), 8.10 (br d, *J* = 7.6 Hz, 2H), 7.65–7.56 (m, 3H), 7.07 (s, 1H), 6.88 (d, *J* = 2.0 Hz, 1H), 6.47 (d, *J* = 2.0 Hz, 1H), 5.43 (d, *J* = 4.8 Hz, 1H), 5.15 (d, *J* = 4.8 Hz, 1H), 5.09 (d, *J* = 4.8 Hz, 1H), 5.08 (d, *J* = 8.0 Hz, 1H), 4.62 (t, *J* = 5.2 Hz, 1H), 3.70 (m, 1H), 3.50–3.43 (m, 2H), 3.32–3.14 (m, 3H). ^13^C NMR (100 MHz, DMSO‐*d*_6_): δ (p.p.m.) = 182.3, 163.9, 163.3, 161.0, 157.3, 132.5, 130.7, 129.4, 126.7, 105.8, 105.6, 100.0, 99.8, 95.3, 77.2, 76.3, 73.1, 69.6, 60.6. ^1^H NMR (400 MHz, DMSO‐*d*_6_, exchanged with D_2_O): δ (p.p.m.) = 8.07 (dd, *J* = 7.2, 2.0 Hz, 2H), 7.64–7.55 (m, 3H), 7.01 (s, 1H), 6.87 (d, *J* = 2.0 Hz, 1H), 6.47 (d, *J* = 2.0 Hz, 1H), 5.06 (d, *J* = 8.4, 1H), 3.69 (m, 1H), 3.48–3.42 (m, 2H), 3.30 (t, *J* = 8.4 Hz, 1H), 3.25 (t, *J* = 8.4 Hz, 1H), 3.16 (t, *J* = 8.4 Hz, 1H). HRMS (ESI‐TOF): *m*/*z* calcd. for C_21_H_20_NaO_9_ [M + Na]^+^ 439.1000, found 439.0997.

### LDL uptake assays

Huh‐7 cells were incubated with 10‐μg·mL^−1^ DiI‐labeled LDL for 2.5 h. The cells were subsequently washed with phosphate‐buffered saline and fixed with 4% paraformaldehyde. Intracellular fluorescent staining was visualized using a fluorescence microscope. The fluorescence intensity was quantified by image j software (Rasband, WS, National Institutes of Health, Bethesda, MD, USA).

### Real‐time quantitative PCR

Total RNA was extracted from Huh‐7 cells using RNAisoPlus (TAKARA, Shiga, Japan), according to the manufacturer's instructions; cDNA was synthesized and amplified from 0.5 μg of the total RNA using a ReverTra Ace® qPCR RT Master Mix with gDNA Remover (TOYOBO, Osaka, Japan). Quantitative real‐time PCR (SYBR green; Roche, Basel, Switzerland) analyses were performed using an Applied Biosystems StepOnePlus Real‐Time PCR System (Foster City, CA, USA). Expression was normalized to glyceraldehyde‐3‐phosphate dehydrogenase (*GAPDH*) control. The following were the sequences of the primer set used: human *LDLR*, 5′‐CAGAGGCAGAGCCTGAGTCA‐3′ and 5′‐CGGGTGTCTCAGGCACTTAA‐3′; human *FAS*, 5′‐CCATCTACAACATCGACACCA‐3′, and 5′‐CTTCCACACTATGCTCAGGTAG‐3′; human *GAPDH*, 5′‐ACCCACTCCTCCACCTTTGA‐3′, and 5′‐CTGTTGCTGTAGCCAAATTCGT‐3′.

### Plasma membrane damage determination

The plasma membrane damage of Huh‐7 cells was determined by LDH assay (Dojindo, Kumamoto, Japan). Huh‐7 cells were plated in 96‐well plates at a density of 2.0 × 10^4^ cells/well and were cultured in medium A for 24 h. After incubation for another 24 h in the absence or presence of 30‐μm chrysin‐7G, LDH assay was performed according to the manufacturer's instructions.

### Antibodies

Polyclonal anti‐LDLR antibody was purchased from Proteintech (Rosemont, IL, USA). Monoclonal anti‐phospho‐AMPKα (Thr172) (40H9), anti‐phospho‐ACC (Ser79) (D7D11), anti‐ACC (C83B10), and anti‐β‐actin (8H10D10) antibodies and polyclonal anti‐AMPKα, anti‐phospho‐ERK1/2 (Thr202/Tyr204), and anti‐ERK1/2 antibodies were purchased from Cell Signaling Technology (Danvers, MA, USA). Peroxidase‐conjugated affinity‐purified goat anti‐rabbit and goat anti‐mouse IgGs were purchased from the Jackson Immunoresearch Laboratories (West Grove, PA, USA). All antibodies were used at a dilution of 1 : 1000.

### Immunoblotting

Cells were lysed in RIPA buffer containing 50‐mm Tris–HCl (pH 7.6), 150‐mm sodium chloride, 0.1% (w/v) SDS, 0.5% (w/v) deoxycholate, 1% (v/v) Triton X‐100, and protease inhibitors. Lysates were subjected to SDS/PAGE; proteins were transferred onto a PVDF membrane and were probed with the indicated antibodies. Immunoreactive proteins were visualized using Western blotting detection reagents, including ECL (Cytiva, Marlborough, MA, USA). Signals on membranes were detected and quantified using AE‐6981 light capture (ATTO, Tokyo, Japan). The signal intensity was quantified by image j software.

### Statistical analysis

All data were presented as mean ± standard error of the mean. Pairwise comparisons of treatments were made using the Student's *t*‐test. Multiple comparisons were made using one‐way analysis of variance followed by the Tukey's *post‐hoc* test. *P* < 0.05 were considered statistically significant.

## Conflict of interest

The authors declare no conflict of interest.

### Peer review

The peer review history for this article is available at https://www.webofscience.com/api/gateway/wos/peer‐review/10.1002/2211‐5463.13665.

### Author contributions

JI coordinated the project. NK, TF, SH, KF, and MK performed the experiments. NK, TF, SH, KF, and JI analyzed the data. SW, RK, KI, TS, YY, and JI were involved in the conception of the research ideas, data analysis, and result interpretation. JI wrote the manuscript. All authors approved the final version of the manuscript.

## Data Availability

The data that support the findings of this study are available from the corresponding author, JI, upon reasonable request.
